# Mec1/ATR, the Program Manager of Nucleic Acids Inc.

**DOI:** 10.3390/genes8010010

**Published:** 2016-12-28

**Authors:** Wenyi Feng

**Affiliations:** Department of Biochemistry and Molecular Biology, SUNY Upstate Medical University, 750 East Adams Street, Syracuse, NY 13210, USA; fengw@upstate.edu; Tel.: +1-315-464-8701

**Keywords:** Mec1/ATR, replication–transcription conflict, checkpoint, DNA damage response, stress response, R-loop

## Abstract

Eukaryotic cells are equipped with surveillance mechanisms called checkpoints to ensure proper execution of cell cycle events. Among these are the checkpoints that detect DNA damage or replication perturbations and coordinate cellular activities to maintain genome stability. At the forefront of damage sensing is an evolutionarily conserved molecule, known respectively in budding yeast and humans as Mec1 (Mitosis entry checkpoint 1) and ATR (Ataxia telangiectasia and Rad3-related protein). Through phosphorylation, Mec1/ATR activates downstream components of a signaling cascade to maintain nucleotide pool balance, protect replication fork integrity, regulate activation of origins of replication, coordinate DNA repair, and implement cell cycle delay. This list of functions continues to expand as studies have revealed that Mec1/ATR modularly interacts with various protein molecules in response to different cellular cues. Among these newly assigned functions is the regulation of RNA metabolism during checkpoint activation and the coordination of replication–transcription conflicts. In this review, I will highlight some of these new functions of Mec1/ATR with a focus on the yeast model organism.

## 1. Introduction

Mec1/ATR (Mitosis entry checkpoint 1 and Ataxia telangiectasia and Rad3-related protein)—an evolutionarily conserved protein in *Saccharomyces cerevisiae* and *Homo sapiens*, respectively—is virtually ubiquitous in all cellular compartments, dispatching work forces to perform a wide range of tasks. The biochemical function of Mec1/ATR is a kinase acting in a complex with Ddc2 (ATRIP, ATR interacting protein in humans) that controls a signaling cascade to ensure the maintenance of genome integrity. Mec1/ATR’s responsibilities are rooted in DNA metabolisms including replication, repair, and chromosome segregation. Growing evidence also extends Mec1/ATR’s function to RNA metabolisms and, more importantly, implicates Mec1/ATR in resolving conflicts between DNA replication and gene transcription. Mec1/ATR’s function is thus akin to that of a Program Manager in an organization, who serves a strategic role by coordinating teams working on related projects. For these reasons, it is appropriate to bestow such a title upon Mec1/ATR in this coined enterprise (and a feeble attempt at a double entendre)—Nucleic Acids Incorporated.

*MEC1* and *RAD53* (radiation sensitive mutant 53, human *CHEK2*) were initially identified in *Saccharomyces cerevisiae* as genes required for the S and G2 checkpoints that are induced by DNA replication inhibition and DNA damage, respectively [1]. When replication inhibition is induced through treatment with hydroxyurea (HU)—a ribonucleotide reductase (RNR) inhibitor—Mec1 (acting as a sensor) phosphorylates a cluster of residues in the N-terminus of Rad53 (a transducer), which in turn activates downstream effector molecules to play multiple cellular functions [2]. A defective checkpoint via mutations in the Mec1 or Rad53 kinase causes cells to lose control over replication initiation, prevent stalled replication forks from resuming progression, prematurely enter mitosis, and ultimately, lose viability [2]. Along with these two kinases, the Tel1 kinase (human ATM, ataxia telangiectasia mutated) partially substitutes Mec1 during its absence, albeit the two kinases show distinct substrate specificities [3]. A recent study provided structural insights into how the Mec1•Ddc2 dimer and Tel1 dimer function differentially towards substrate [4]. The kinase domains within the Mec1•Ddc2 dimer are quite close to each other, thus potentially requiring a dimer-to-monomer structural change to activate the kinase. In contrast, the kinase domains within the Tel1 dimer are sufficiently far from each other, thus permitting the kinase activity even in the dimer configuration. This structural difference may help explain the substrate specificity of the ATR and ATM protein in specific pathways.

Though premature mitosis is a prominent phenotype associated with the checkpoint mutants upon replication stress [1,5], restraining mitosis apparently does not ameliorate the loss of cell viability and, instead, the essential function of the checkpoint appears to be promoting recovery from stress [6]. Moreover, cell lethality associated with *mec1* and *rad53* deletions can be rescued by the removal of a protein inhibitor of RNR, Sml1, suggesting that nucleotide pool maintenance is the underpinning for Mec1/Rad53 functions during an unperturbed cell cycle [7]. Thus, it seems that the critical function(s) of the checkpoint pathway vary based on the situation at hand, be it a normal cell cycle or during induced stress. In other words, certain functions of these enzymes only become essential upon induced stress, including nutrient deprivation, external DNA damage, and replication blockage. Incidentally, it is these stress-induced functions of the checkpoint that are better characterized so far. However, recent studies have identified new substrates of the Mec1/ATR in a normal S phase, which promise to further our understanding of the essential function of the checkpoint without external stress. As well, the stress-induced checkpoint functions of Mec1/ATR are an ever-expanding list. Here, I will focus on reviewing the roles that Mec1/ATR play in nucleic acid metabolisms, including DNA replication, gene transcription, and the interface between these processes. I will also highlight recent studies presenting Mec1/ATR’s new functions in various cellular pathways, with a focus on the model organism *Saccharomyces cerevisiae*. 

## 2. The To-Do List of Mec1/ATR in DNA and RNA Metabolisms

### 2.1. DNA Metabolism

#### 2.1.1. Nucleotide Pool Maintenance

Following DNA damage or replication blockage, the Mec1/Rad53 checkpoint induces the Dun1 kinase, which in turn down-regulates Sml1 (an inhibitor of RNR), thus allowing a boosted production of deoxyribonucleotides (dNTPs) [8]. Later, it was demonstrated that this signaling pathway is also crucial during unchallenged cell growth. As mentioned above, the lethality associated with *mec1* or *rad53* deletion can be suppressed by increasing RNR activity through removal of its protein inhibitors, including *sml1*, *crt1*, *hug1*, and *dif1*, or overexpressing *RNR1* or *RNR3* [6,7,9,10,11,12]. Therefore, it appears that the essential function of the Mec1/Rad53/Dun1 checkpoint cascade during normal growth is to maintain a proper level of dNTP pools. Indeed, the dNTP levels are depleted—with a broad range of depletion levels—in various *mec1*, *rad53*, and *dun1* mutants compared to wild-type yeast [9,13,14].

The mechanisms by which Mec1/Rad53 signaling ensures an adequate and balanced pool of dNTPs largely centered on RNR regulation. RNR activity can be turned on/off by its binding to ATP or dATP (deoxyadenosine triphosphate), respectively, at an allosteric activity site [15,16]. It is also subject to direct transcriptional induction and indirect upregulation by the destruction of the aforementioned protein inhibitors, both through the Mec1/Rad53 pathway, as reviewed in [17]. However, what is the molecular consequence of the failure to maintain dNTP levels during normal growth? Using constructs containing regulated expression of Sml1, it was shown that prolonged inhibition of RNR results in a terminal phenotype of incomplete DNA replication in *mec1* and *rad53* deletion mutants [18]. It was also shown that a *mec1-21* mutant is hyper-recombinogenic in an Sml1-dependent manner [14]. In addition, a *rad53-4AQ* mutant that lacks the N-terminal cluster of phosphorylation sites by Mec1 is unable to activate Dun1, and is synthetically lethal with *rad9* without external damage [19]. Together, these data argue that there is a minimal requirement of Dun1 activation by the Mec1/Rad53 checkpoint during a normal S phase to maintain an adequate level of dNTPs, protect cells from DNA damage, prevent hyper-recombination, and ensure complete DNA replication.

The broad range of dNTP pool level reduction in checkpoint mutants is intriguing. In some of the mutants examined, the reduction of dNTP levels was rather modest. Notably, in a *mec1* temperature-sensitive (*mec1^ts^*) lethal mutant, there was only a 17% drop in dNTP pools at the restrictive temperature [13]. It was argued that the *mec1* mutant is exquisitely sensitive to even minute levels of dNTP reduction due to the wide range of DNA metabolic pathways for which dNTPs are required [13]. Therefore, it appears that the next challenge in understanding the essential function of the Mec1/Rad53 checkpoint for normal cell growth is the identification of those checkpoint substrates in a normal cell cycle. Conceivably, although both *mec1* and *rad53* deletion mutants can be suppressed by up-regulating RNR, the respective essential functions of these kinases might differ. Consistent with this notion, a recent phosphor-proteomic screen has revealed >200 peptide substrates represented by genes of the Mec1/Tel1 kinases during normal S phase. Approximately 50% of these substrates are Rad53-independent, and their phosphorylation is not further induced by HU or DNA damage by methylmethane sulfonate (MMS) [20]. Therefore, it stands to reason that these protein substrates of Mec1/Tel1 might define the essential function of the Mec1 kinase during normal growth (more on this later). 

#### 2.1.2. Regulation of Origins of Replication and Replication Forks

As alluded to above, the essential function of the Mec1/Rad53 checkpoint may vary depending on the growth conditions. During replication stress, it is thought that the checkpoint is essential for the preservation of the integrity of the replication fork, facilitated by maintaining a critical level of dNTP pools. It was also demonstrated that HU- or MMS-treated *mec1* or *rad53* cells fail to inhibit late origin activation, which is considered as another underlying cause of cell death [21,22]. Consistently, checkpoint-deficient cells that sustained irreparable UV damage also activate late origins prematurely during DNA synthesis and lose viability [23]. Together, these studies cemented the notion that premature origin activation during replication stress is detrimental to genome stability. Subsequent studies have revealed mechanisms through which the checkpoint imparts an inhibitory signal to late origins: via two initiation factors—Sld3 and Dbf4 proteins—that are subject to phosphorylation by Mec1/Rad53 and the Cdc7 kinase, respectively [24,25]. It was shown that regulatory domains of the Mcm4 helicase subunit also play a role in the control of late origin activation [26]. A recent study that combined mutations in all three substrates (Sld3, Dbf4, and Mcm4)—rendering them refractory to checkpoint control—showed a global activation of late origins at a similar level as that in the *mec1* or *rad53* checkpoint mutant [27]. However, the late origin activation phenotype in the triple mutant is only elicited by HU treatment, suggesting that the level of premature late origin activation is not critical enough to jeopardize cell viability during normal growth. 

The unrestrained late origin firing in checkpoint mutants during replication stress is accompanied by defective replication fork progression. The function of Mec1/ATR at stalled replication forks during replication stress has been the subject of several comprehensive reviews [28,29,30,31,32]. It is now generally accepted that the absence of checkpoint functions leads to a global level of replication fork collapse, such that the forks are not capable of resumption following the removal of replication stress. The molecular insight into the anatomy of a “collapsed replication fork” was first provided by a seminal study demonstrating extensive single-stranded DNA (ssDNA) accumulation at the replication fork in a *rad53-K227A* kinase-deficient mutant following replication stress by HU [33]. Concurrently, it was shown by another influential study that conditionally lethal *mec1* mutants exhibit breakage at specific regions of the chromosome, akin to the formation of mammalian chromosome fragile sites [34]. Subsequent studies confirmed the formation of fork-associated ssDNA in both *rad53* and *mec1* mutants upon replication stress, and provided genomic views of the ssDNA at origins of replication [35,36]. It was also shown that the ssDNA, when bound by the eukaryotic ssDNA-binding protein RPA (Replication protein A), constitutes the signal to recruit Mec1/ATR to the replication fork and trigger the signaling cascade [37]. Moreover, it was demonstrated that ssDNA formation at a replication fork destines the fork to DNA double strand breaks and fragile site formation, as previously seen in the *mec1* conditional mutant [38].

**Figure 1:**
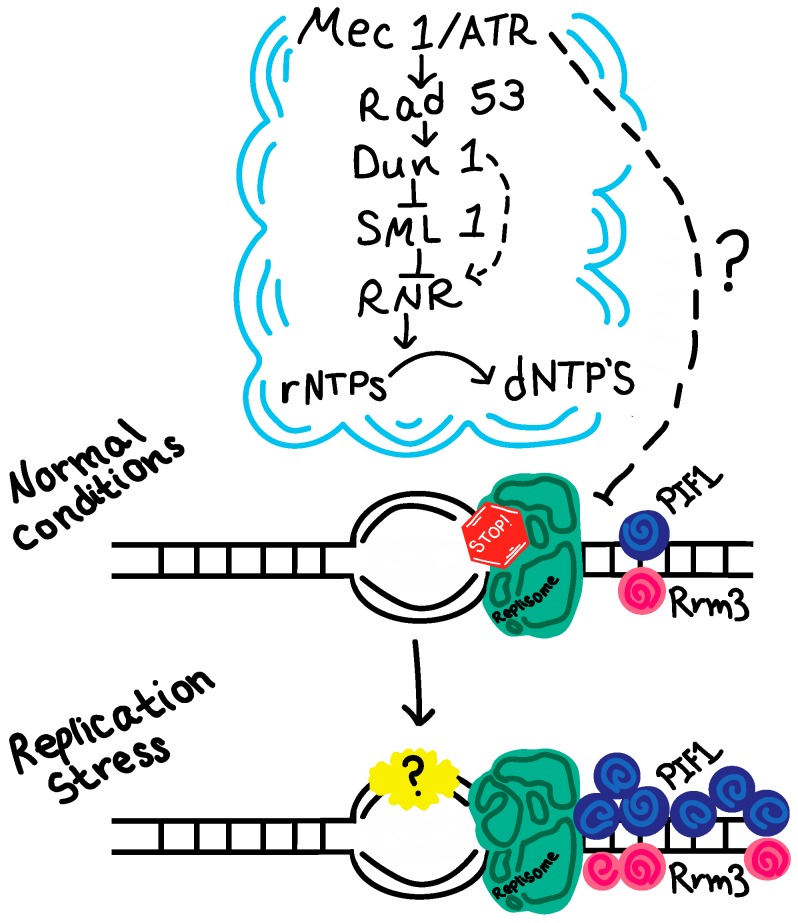
Schematic representation of replication fork protein dynamics during replication stress. A recent study showing the differential retention of two stressed fork-associated helicases, Pif1 and Rrm3, is featured here. The question mark denotes what future investigation should aim to reveal—potential dynamic changes occurring behind the fork during replication stress. rNTP: ribonucleotide; dNTP: deoxyribonucleotide; Mec1/ATR: Mitosis entry checkpoint 1/ataxia telangiectasia and Rad3-related protein; Rad53: radiation sensitive mutant 53; Dun1: DNA damage uninducible 1, transcriptional inhibitor of *SML1*; *SML1*: suppressor of *MEC1* lethality 1, inhibitor of RNR; RNR: ribonucleotide reductase.

However, the exact nature of the protein composition and possible transformation at the collapsed fork is still not clear. Previous studies have suggested that replisome stability is compromised in checkpoint mutants when the replication fork is impeded [39,40]. In contrast, recent evidence argues that the replisome components are largely intact in both *mec1* and *rad53* mutants [41]. Interestingly, in HU-treated human cells, ATR inhibition resulted in genome instability without destabilizing the replisome, but instead involved altered recruitment of other fork-associated proteins [42]. Consistent with this notion, a recent yeast study demonstrated that two DNA helicases involved in replication fork restart—Rrm3 or Pif1—are differentially clustered at replication forks, with a higher retention of Pif1 than Rrm3 [43]. Moreover, removal of either Pif1 or Rrm3 rescues cell lethality in *rad53* cells treated with HU [43]. These observations demonstrated an altered architecture of the replication fork during replication stress, and suggested that the restoration to the normal architecture is key to the maintenance of a stalled fork. Rrm3 and Pif1 are both regulated by Mec1/Rad53–mediated phosphorylation, and a phosphor-mimic *rrm3-6SD* mutant can rescue the phenotypes of the *rad53* mutant during replication stress [43]. It would be interesting to test if the altered recruitment of fork-associated proteins is also recapitulated in yeast. It would also be important to determine what dynamic changes might occur behind a stressed replication fork (Figure 1). Future studies could be directed towards comparative analysis of the full architecture of a stalled replication fork vs. a normal one in yeast by capitalizing on a mini-chromosome purification system previously described [44] or similar methods.

### 2.2. RNA Metabolism

#### 2.2.1. RNA Processing during Damage-Sensing in the Checkpoint Pathway

Activation of the Mec1/ATR kinase involves recruitment of the protein to the chromatin, at DNA double strand break (DSB) sites [45,46], at stalled replication forks [37], or at shortened telomeres [47,48]. In the cases of DSBs and stalled forks, RPA-coated ssDNA activates Mec1/ATR, making DNA intermediates the key molecule at the center of checkpoint signaling. However, increasing evidence also places RNA molecules in this pathway. Indeed, there is a clear interplay between pre-mRNA processing and the checkpoint response in metazoans, as reviewed [49].

How does RNA processing play a role in damage sensing of a checkpoint response? It was recently shown that the RNA decay factors in yeast—Xrn1, Rrp6, and Trf4—are important for DSB-sensing as they promote the formation of RPA-ssDNA [50]. The precise function of these RNA processing factors at the damaged DNA site is unknown. Clearing the DNA template for possible DNA:RNA hybrid molecules—also known as the co-transcriptional R loops—does not appear to be the reason, because increased production of RNase H1 (which degrades DNA:RNA hybrids) does not promote RPA-ssDNA formation in the absence of Rrp6 or Trf4 [50]. However, this result does not exclude the possibility that RNA molecules in contexts other than DNA:RNA hybrid might be responsible for the blockage of RPA-ssDNA formation. For instance, an aberrant mRNP (messenger ribonucleoprotein) particle may be obstructing the damaged site. This hypothesis is substantiated by recent findings that Rrp6 plays an important role in the quality control of specific mRNPs [51,52]. Moreover, a previous proteomic analysis revealed a multitude of interactions between RPA and the chromatin remodeling proteins, including Ino80, Isw1, Isw2, Swic, Rsc2, and SWI/SNF [53]. The question then becomes “do these proteins play a role in promoting RPA-ssDNA formation at the damaged fork?” Indeed, there has been some evidence suggesting that in mammalian cells, chromatin remodeling factors such as INO80 facilitate RPA-ssDNA formation during DSB processing [54,55]. Finally, it would also be interesting to determine if RNA processing plays a role in the detection of RPA-ssDNA in the context of a stalled fork.

#### 2.2.2. Transcription Regulation in cis of DNA Damage or Stalled Forks 

It has been documented that in mammalian cells, both RNA Pol I- and Pol II-mediated transcriptional silencing/inhibition occurs in the vicinity of damaged DNA (e.g., at induced DSBs), in an ATM-dependent manner [56,57,58]. Similarly, ATR is responsible for transcription repression at clusters of stalled replication forks induced by doxorubicin [59]. However, this checkpoint-dependent transcriptional inhibition response is contentious in yeast, at least at an induced DSB [60]. Yet, in both mammalian cells and yeast, Mec1/ATR and Tel1/ATM phosphorylate histone H2AX at a C-terminal serine to generate gamma-H2AX, thereby causing changes in the chromatin environment at the damaged DNA site [61,62,63,64]. Whether this chromatin remodeling is the cause of transcription inhibition or merely the reflection of the latter is not clear. Therefore, the exact role of the checkpoint in transcription silencing at a DSB site still warrants further investigation. 

Proteomic studies in mammalian and yeast systems both identified components of the hnRNP (heterogeneous nuclear ribonucleoprotein) complex as substrates of the Mec1/ATR kinase during replication stress [65,66]. A recent study identified 115 peptides, represented by 71 genes, as Mec1/Tel1- and Rad53-dependent substrates during replication stress [20]. The molecular functions of these genes are enriched in DNA replication and response to DNA damage, as expected [20]. In addition, this gene group is also enriched for those in regulation of transcription [GO:6355], chromatin silencing [GO:6348, 30466], and mRNA transport [GO:51028] (*p* = 9.44 × 10^−7^, 2.39 × 10^−6^, 3.15 × 10^−5^, and 3.25 × 10^−5^, respectively). Moreover, as mentioned earlier, 117 peptides represented by 81 genes were identified as Mec1/Tel1-dependent and Rad53-independent substrates in normal S phase, and they are highly enriched for genes involved in transcription, chromatin remodeling, and RNA processing [20]. These findings therefore invite the hypothesis that Mec1/Tel1/Rad53 checkpoint proteins play a role in regulating gene transcription and related activities both during normal DNA synthesis and upon replication stress (see more below).

### 2.3. The Interface between DNA and RNA Metabolism—Resolving Replication and Transcription Conflicts

The functions of Mec1/ATR in the pathways described above naturally necessitate the checkpoint function at the junction between DNA replication and gene transcription, which share the same chromosome template. Indeed, these two processes can be in physical conflict when the DNA and RNA polymerase complexes are stalled for various reasons [67]. Notably, a progressing replication fork can encounter a Pol II complex blocked by stable R-loop formation. As alluded to before, R-loops are co-transcriptional structures defined by a hybrid between the nascent RNA transcript and one of the DNA template strands, leaving the other DNA strand exposed as single-stranded [68,69,70,71]. It is thought that stable R-loop formation could impede the replication machinery, triggering both homologous recombination and non-homologous end-joining, suggesting that these sites have undergone DSB formation [72,73,74,75,76,77,78]. Thus, stable R-loop formation impedes replication forks and is a detriment to genome stability. 

Replication–transcription conflicts can also originate from a defective replication fork encountering unscheduled transcription activities, particularly during induced replication stress. My laboratory recently showed that the replication inhibitor HU can simultaneously stall replication forks and induce unscheduled gene expression, leading to chromosome breakage [79]. Our study provides an explanation for why different replication inhibitors can produce distinct chromosome breakage patterns—it is the result of differential sites of replication–transcription conflicts dependent on the drug-specific gene expression profiles. Consistent with this notion, it was recently shown that estrogen-induced DSBs occur where replication encounters estrogen-responsive genes [80]. These studies thus highlighted the importance of understanding the gene expression profiles of replication inhibitors, which are widely present in the environment and are commonly used in medical practices (e.g., anti-cancer drugs).

**Figure 2:**
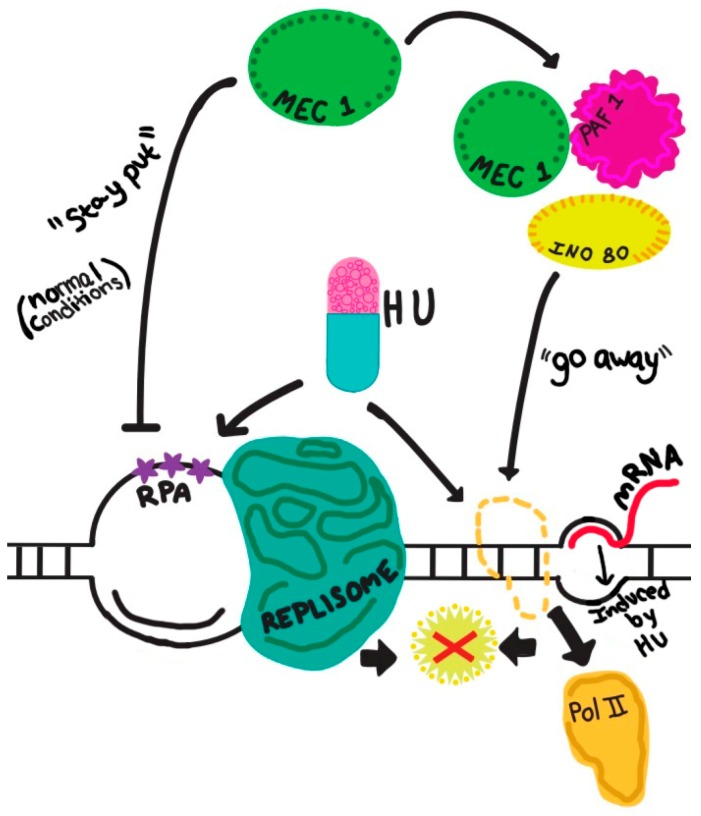
Schematic representation of converging replication and transcription, endangering the chromosome template for DNA double strand breaks (depicted by a red cross). The dual effects of a replication inhibitor, (e.g., hydroxyurea, HU) simultaneously impacting replication and transcription (shown by two arrows descending from “HU”) are described in the main text. The precise function of Mec1 in the protection of a stressed (e.g., by HU) fork is yet to be defined, and is depicted as a “stay put” signal, which likely also operates during a normal S phase. The inhibitory nature of the signal is sheer speculation at present. The active removal of RNA Pol II by the Mec1-Ino80-Paf1 complex during replication–transcription conflict is featured here. Other novel protein complexes involving Mec1 await future discoveries. RPA: replication protein A, the ssDNA-binding protein.

What is the role of Mec1/ATR in preventing and/or resolving conflicts between replication and transcription? This topic has recently been extensively reviewed [81]. Here I summarize the two broad aspects of Mec1/ATR’s function in this process reported so far: maintaining fork stability as discussed earlier, and eviction of the transcription complex. In the absence of Mec1 (in a *mec1Δ sml1Δ* mutant) the replication fork produces extensive ssDNA at replication forks, and ultimately leads to DSBs [38]. However, does Mec1/ATR also exert any function on the transcription complex? A recent study illuminated the other side of the coin, so to speak, by presenting a novel function of Mec1 in removing RNA Pol II from the template to preserve replication fork integrity when replication and transcription are in conflict [82]. In this specific capacity, Mec1 forms a complex with the chromatin remodeling factors Ino80 and Paf1, where Ino80 serves as a substrate—possibly at Ser51 and Thr568—for Mec1 [82]. It stands to reason that Mec1 can also complex with other proteins to modulate the replication fork proteins during replication–transcription conflict. This study also underscores the importance of proteomic studies in identifying novel Mec1-interacting proteins and checkpoint functions. These findings are depicted in a juxtaposition of DNA replication and transcription approaching each other in a head-on configuration (Figure 2).

One of the hurdles arising from replication–transcription conflict is the torsional stress (positive and negative supercoiling) generated in the chromosomal DNA. Many pathways are involved in the prevention of accumulated DNA torsional stress, including (but not limited to) mRNP biogenesis (THO/TREX complex), template unwinding (Rrm3 helicase), chromatin remodeling (FACT complexes), R-loop prevention (RNase H, topoisomerase I, MCM helicase, etc.), and gene gating (nuclear pore complexes) [83,84,85,86,87,88]. Of note, Mec1/ATR is important for severing the actively transcribed genes from the tethered nuclear pore complex during replication–transcription conflicts, representing yet another solution to protect replication forks [87]. Many proteins in these pathways are substrates of Mec1/ATR [20]. For instance, seven nuclear pore complex proteins (Nup60, Gle1, Yrb2, Mlp1, Nup2, Nup188, and Nup1) and ten chromatin silencing factors (Fun30, Mrc1, Sum1, Ino80, Nup2, Spt21, Esc1, Rlf2, Net1, and Top1) were identified as Mec1/Tel1/Rad53-dependent substrates during replication stress from the study by Bastos de Oliveira et al. In addition, four proteins in the mRNA export pathway (Hpr1, Yra1, Sgf73, and Thp2) are Mec1/Tel1-dependent and Rad53-independent substrates in normal S phase. Mutation studies designed to probe the molecular functions of the checkpoint-dependent phosphorylation of these proteins will shed new light on this pathway.

### 2.4. Other Specialized Tasks

#### 2.4.1. Dealing with Mechanical Stress

The Mec1/ATR-mediated function at the nuclear periphery described above apparently goes beyond the capacity of resolving replication–transcription conflicts. It has been shown that Mec1/ATR also regulates a pathway that senses general stress to the nuclear envelope, produced either by torsional stress of the chromosomal DNA in the processes discussed above, or through osmotic pressure and mechanical force [89]. All these stimuli can increase the location of Mec1/ATR at the nuclear envelope, where it regulates chromatin condensation and nuclear envelope breakdown. It is an exciting property of the Mec1/ATR kinase, and the players involved in this signaling pathway will prove interesting, as mechanical stress was induced without incurring DNA damage. It will also be interesting to determine to what extent the Mec1/ATR substrates in the mechanical stress pathway overlap with those in other stress–response pathways.

#### 2.4.2. Dealing with Nucleolar Stress

Recent studies have demonstrated that ATR/ATM-mediated DNA damage response results in transcriptional regulation and organized DSB repair in the nucleolus, as reviewed in [90]. Indeed, ATM was observed to be localized specifically to the nuclear caps following DNA damage [91]. As a response to nuclear envelope stress described above, ATR was also seen localized in the nucleolus [89]. It has been shown that in response to chromosome breaks, the ATM pathway inhibits Pol I transcription at the ribosomal DNA (rDNA) loci in mouse embryonic fibroblasts [56]. Analogous to the eviction of RNA Pol II by Mec1-Ino80-Paf1, ATM activity was shown to be important for displacing the RNA Pol I elongating complex [56]. Does ATM mediate Pol I transcription directly or through an intermediary? Recent studies showed that ATM signaling following DNA damage triggers Pol I silencing through the interaction between the Nijmegen breakage syndrome (NBS) protein and a nucleolar factor TCOF1-Treacle [92,93]. These studies suggested that the ATM-mediated DNA damage signaling is capable of propagating in-trans through nuclear compartments. The precise mechanism through which nucleolar NBS in conjunction with Treacle causes Pol I inhibition remains to be determined. It is also important to understand the significance of Pol I transcription inhibition in the presence of genomic DNA damage.

#### 2.4.3. Nutrient Sensing

The Mec1 signaling pathway can also cooperate with the nutrient response pathways in yeast. For instance, when cells grown on a non-fermentable carbon source receive glucose, it triggers a transient peak of cyclic adenosine monophosphate (cAMP) production through the Ras pathway, which in turn stimulates the cAMP-dependent protein kinase A (PKA) activity and drives S phase progression [94]. It is thought that this PKA response is also important for restraining mitosis if the daughter cell has not reached a critical size [95]. This negative regulation of cell cycle progression by PKA is apparently exploited by the Mec1-mediated checkpoint pathway in response to DNA damage [96]. Subsequent investigation revealed that Mec1 directly phosphorylates the regulatory subunits of PKA, thereby activating the catalytic subunit of the kinase [97]. Similar to this partnership with the PKA pathway, Mec1/Tel1 can also draw on other substrates in the glucose-sensing pathway, such as Snf1 (the AMP-dependent kinase), and by down-regulating Snf1 steer cells towards aerobic fermentation instead of respiration [98]. It was proposed that the Mec1-mediated DNA damage response produces a cellular decision analogous to the Warburg effect in cancer cells [98].

## 3. Concluding Remarks

The vast range of the Mec1/ATR-mediated signaling pathways precludes a thorough coverage in this review. Here I highlighted some of the recent studies describing Mec1/ATR’s roles in nucleic acid metabolisms. From these studies, we can glean several key features of the Mec1/ATR protein function. First, it appears that Mec1/ATR functions modularly and complexes with different proteins in specific cellular contexts to exploit existent pathways, or to acquire new functions. Second, RNA molecules play increasingly more complex roles in chromosomal DNA transactions. With the discovery of new substrates of the Mec1/ATR during a normal cell cycle, or when cells are under stress, we will continue to discover new genes and molecules in the crossroads of DNA replication and gene expression. Finally, there are certain topics in the Mec1/ATR-associated biology that are not covered in detail here. For instance, the list of activators of Mec1/ATR continues to expand, one of the latest examples being that the SWI/SNF chromatin remodeling complex specifically regulates Mec1 kinase activity during S phase, independent of the known regulators of Mec1 such as Dpb11 [99]. Though the precise function of this regulation is not clear, one can envisage yet another layer of complexity in the Mec1-mediated pathways in response to the SWI/SNF-regulated processes.
